# The path mechanism of occupational burnout affecting the health of emergency nurses: a moderated mediation model of work–family behavior role conflict and the work environment

**DOI:** 10.3389/fpubh.2026.1791032

**Published:** 2026-04-02

**Authors:** Chuanxi Chen, Xiaoli Chen, Hao Zhang, Lin Lan, Luying Zhong, Min Dai, Yu Cao

**Affiliations:** 1 Department of Emergency Medicine, West China Hospital, Sichuan University, Chengdu, China; 2 Laboratory of Emergency Medicine, West China Hospital, Sichuan University, Chengdu, China

**Keywords:** emergency nurses, occupational burnout, occupational health, work environment, work-family behavior role conflict

## Abstract

**Objectives:**

The purpose of this study is to discover how occupational burnout influences the health status of emergency nurses and to investigate the mediation effect of work–family behavior role conflict.

**Design:**

A multi-center cross-sectional study.

**Setting and participants:**

A questionnaire survey of 1,540 emergency nurses from 30 tertiary hospitals in China was conducted between December 26, 2023, and January 18, 2024.

**Methods:**

Using an online questionnaire, we performed a cross-sectional survey to collect demographic data and information about occupational burnout, work environment, work–family behavior, role conflict, and emergency nurses’ health status. The PROCESS macro for SPSS 26.0 was used to analyze the moderated mediation model, and the bootstrap approach was used to investigate the mediating effects.

**Results:**

The findings revealed that 57.3% of the nurses had experienced occupational burnout. A substantial positive association was identified between professional burnout, work–family behavior role conflict, and somatic symptoms (*r* = 0.493, 0.534; *p* < 0.001). Occupational burnout was found to be a significant predictor of somatic symptoms, with work–family conflict serving as a mediator (*β* = 0.616, *t* = 13.295, *R*^2^ = 0.488, *p* < 0.01). Our research found that nurses’ work environment mediated the association between burnout and work–family behavior role conflict (*β* = 0.007, *t* = 3.647, *p* < 0.01), indicating that a positive work environment may reduce the impact of burnout on family role conflict.

**Conclusion:**

Work–family behavioral role conflict and the nursing work environment were found to partially mediate the association between occupational burnout and the health outcomes of emergency nurses. These findings suggest that, while interventions aimed at mitigating work–family role conflict and improving the work environment are essential, they may not be sufficient on their own to safeguard nurses’ health. Additional strategies are needed to comprehensively address the health risks associated with occupational burnout. Moreover, the interplay among burnout, work–family conflict, and environmental factors underscores the necessity of integrated and multifaceted intervention approaches to alleviate the health burden experienced by emergency nurses effectively.

## 1 Introduction

Occupational burnout among healthcare personnel has become a major public health concern (1, 2). Nursing professionals are also at significant risk of occupational burnout, with studies indicating that roughly 30.7% of nurses worldwide experience burnout (3). A statewide survey of 51,406 nurses in China found that roughly 50% experienced burnout (4). Burnout triggers a wide range of physical and mental health problems, including anxiety, depression, sleep disorders, and cardiovascular diseases (5–7). Furthermore, burnout impairs nurses’ job performance, increasing the likelihood of errors and adverse events (8, 9). Although the association between burnout and poor mental health is well recognized, previous research has focused on connections between burnout and mental health across populations, with little emphasis on the pathways or processes by which burnout impacts nurses’ health. As a result, the fundamental mechanisms by which occupational burnout affects nursing populations’ health are unknown and debated. Occupational burnout negatively predicts the health status of emergency nurses. Occupational burnout negatively predicts the health status of emergency nurses.

Work–family conflict functions as a unique job demand in the modern workplace. Both work and family domains have a significant impact on the association between occupational stress and nurses’ physical and mental health, as work–family balance is an important health protection. Some researchers contend that gender variations exist in the association between professional burnout and unfavorable mental health consequences (10). High job demands often deplete the time and energy resources of emergency nurses, thereby precipitating work-to-family conflict. Conversely, family resources (e.g., emotional support from partners, stable family financial situations, and flexible household role arrangements) can effectively mitigate family-to-work interference, enabling nurses to concentrate more on their professional duties and thus buffering family-to-work conflict. Its negative impacts on nurses include psychological stress, depression, anxiety, and the development of harmful habits that affect physical and mental health (11). Furthermore, it contributes to emotional weariness and lower subjective well-being among healthcare professionals (12). Previous research has primarily focused on work-related factors such as occupational burnout to explain how workplace variables affect nurses’ health outcomes, often ignoring the influence of family-domain variables. This study takes into account characteristics from both the work and family domains to elucidate the role of work–family conflict in mediating the association between occupational burnout and emergency nurses’ physical–mental health.

The Job Demands-Resources (JD-R) model posits that job resources, including autonomy and social support, play a crucial buffering role in the relationship between job demands and strain (13). Nursing work environment refer to the factors that assist nurses in achieving work goals, mitigating the depletion caused by job demands, and fostering their professional growth (14). Work stress, according to the person–environment fit model, is caused by a mismatch between an individual’s abilities and the expectations of their workplace (15). According to research, the nursing work environment may play a mediating role in the association between job-related stress and nurse health outcomes (16–18). A healthy nursing work environment improves care quality, increases job satisfaction, and helps to reduce occupational burnout (19–21). However, Havaei et al.’s (22) study found that exposure to job stressors in healthy work settings was associated with increased reports of physical and mental health difficulties among nurses, resulting in conflicting findings regarding the work environment moderating effect. Furthermore, it is unclear if the moderating effect of the nursing work environment on the connection between occupational burnout and nurse health is influenced by family-related variables.

Using the JD-R Model, as the theoretical framework, we integrated both family-related and organizational factors to develop a moderated mediation model, with the aim of elucidating the mechanisms through which occupational burnout impacts the health of emergency nurses. In particular, we examined the mediating role of work–family behavioral role conflict and the moderating effect of the nursing work environment while also considering the potential buffering influence of job resources.

## 2 Methods

### 2.1 Study design

This study is part of a multi-center, cross-sectional survey conducted among 1,540 emergency nurses across China. The primary focus of the survey was to examine the health status of emergency nurses in relation to their work and family characteristics. The study adheres to the Strengthening the Reporting of Observational studies in Epidemiology (STROBE) guidelines.

### 2.2 Research participants

A stratified cluster sampling method was employed based on China’s geographical regions (Northeast, North China, Central China, South China, Southwest, Northwest and East China) to recruit participants. The study encompassed emergency nurses from 30 tertiary hospitals. The hospital distribution was as follows: four in Northeast China, four in North China, four in Central China, five in South China, four in Southwest China, four in Northwest China, and an additional five in East China. Eligible participants met the following criteria: (1) aged ≥18 yr., (2) had at least 1 yr. of emergency nursing experience. A power analysis (*α* = 0.05, power = 80%) based on preliminary data indicated that a minimum of 1,330 participants was required (23). Considering a dropout rate of 10%, the minimum sample size is estimated to be 1,477 emergency nurses.

### 2.3 Research instruments

#### 2.3.1 General information questionnaire

This questionnaire, developed by the research team, collected demographic information, including age, gender, years of work experience, frequency of night shifts, personal monthly income, marital status, educational attainment, reproductive status.

#### 2.3.2 Occupational burnout survey

Occupational burnout was measured using the Chinese version of the Maslach Burnout Inventory–General Survey (MBI-GS), revised by Zhu et al. (24). The scale comprises 16 items across three dimensions: emotional exhaustion, depersonalization, and personal accomplishment. Emotional exhaustion and depersonalization are scored positively, while reduced personal accomplishment is reverse-scored. The composite burnout score is calculated as follows: 0.4 × Emotional Exhaustion + 0.3 × Depersonalization + 0.3 × (6 − Personal Accomplishment). Burnout levels are classified as follows: <1.5 = no burnout, 1.5–<3.5 = suspected burnout, and ≥3.5 = burnout. Cronbach’s *α* values for the scale was 0.860. In this study, the Cronbach’s α coefficient for the scale was 0.904.

#### 2.3.3 Nursing work environment scale

The Nursing Practice Work Environment Scale, developed by Shao et al. (25), was used to assess perceptions of the work environment. The 26-item scale comprises seven dimensions: professional development, leadership and management, physician–nurse relationships, recognition atmosphere, professional autonomy, basic guarantees, and adequate staffing. Items are rated on a 6-point Likert scale (1 = *strongly disagree*; 6 = *strongly agree*), with higher scores indicating a more positive work environment. The Cronbach’s *α* coefficient of the scale is 0.981 in this study.

#### 2.3.4 Work–family behavioral role conflict scale

Work–family behavioral role conflict was measured using the Chinese version of the Work–Family Behavioral Role Conflict (WFEBRC) Scale(26). The scale comprises 19 items across two dimensions: work-to-family conflict (WFC) and family-to-work conflict (FWC). Items are rated on a 5-point frequency scale (1 = never; 5 = very frequently), with higher scores indicating greater role conflict. The overall Cronbach’s *α* was 0.95. In this study, the Cronbach’s α coefficient for the Work-Family Behavioral Role Conflict Scale was 0.969.

#### 2.3.5 Emergency nurse health scale

Health status was assessed using the Chinese Self-Reported Somatic Symptom Scale (SSD-CN) developed by Jiang et al. (27). The 20-item scale includes four dimensions: somatic symptoms (10 items), anxiety, depression, and anxiety–depression. The somatic items cover major bodily systems, while the remaining items assess psychological symptoms. Responses are rated on a 4-point Likert scale (1 = *no symptoms*; 4 = *symptoms present almost daily or difficult to endure*). The scale’s diagnostic cutoff is 36 points, with a sensitivity of 0.97 and specificity of 0.96. In this study, the Cronbach’s α coefficient of the scale is 0.974.

### 2.4 Data collection

An electronic questionnaire was created utilizing the Wenjuanxing platform, which contains an informed consent module, and all items were marked as essential to ensure data completeness. To avoid repeated responses, the system allows only one contribution per IP address. Prior to the study’s execution, each hospital’s leaders received identical training to provide precise and consistent explanations of core ideas and directives. During the data collection phase, the questionnaire QR code link was distributed via the WeChat platform, and the researcher described the research aims to the participants. After reading the informed consent form and agreeing to it, users entered the answering interface. If they have any questions during the answering process, they can contact the training leader at their hospital.

### 2.5 Statistical analysis

Statistical analyses were conducted using SPSS version 26.0. Normally distributed continuous variables were reported as means ± standard deviations. Pearson correlation analysis was used to examine bivariate relationships among the measured variables. The PROCESS macro for SPSS (49) was employed to estimate direct, indirect, and moderated mediation effects. This approach allows for the simultaneous analysis of relationships among multiple variables.

First, to test the mediating role of work–family conflict, we applied PROCESS Model 4. In this model, job burnout was specified as the independent variable (X), work–family conflict as the mediator (M), and health outcomes (e.g., somatic symptoms, psychological well-being) as the dependent variable (Y). Model 7 is a moderated mediation model that tests whether the effect of the independent variable (job burnout) on the mediator (work–family conflict) is conditional upon a moderator (nursing work environment). The significance of the effect was assessed using a bias-corrected bootstrap method with 5,000 samples to generate a 95% confidence interval (95% CI).

### 2.6 Ethical considerations

This study received ethical approval from the Ethics Review Committee of West China Hospital, Sichuan University (Approval No: 2024.309). All research subjects provided informed consent and voluntarily participated in the study.

## 3 Results

### 3.1 Characteristics of participants

A total of 1,540 emergency nurses were surveyed. The mean age was 32.23 ± 6.80 yr. (range: 20–58 yr.). Among them, 329 (21.4%) were male, and 1,211 (78.6%) were female. The majority were married (*n* = 980, 63.6%) and held a bachelor’s degree or higher (*n* = 1,354, 87.9%); 830 (53.9%) reported having children. Of all respondents, 882 (57.3%) reported experiencing occupational burnout. Detailed sociodemographic information, work characteristics, and lifestyle data are presented in Table 1.

**Table 1:** Characteristics of the participants (*n* = 1,540).

Variable	Category	*n*	%
Age	32.23 ± 6.79(years old)		
Gender	Female	1,211	78.60
	Male	329	21.40
Marital status	Unmarried	560	36.40
	Married	980	63.60
Reproductive status	No	710	46.10
	Yes	830	53.90
Educational level	Associate degree	186	12.10
	Bachelor’s degree or above	1,354	87.90
Professional title	Junior RN	907	58.90
	Middle RN	574	37.30
	Senior RN	59	3.80
Years of nursing experience	≤5 years	491	31.90
	6–10 years	493	32.00
	11–15 years	300	19.50
	16–20 years	121	7.80
	>20 years	135	8.80
Working hours per week	≤40 h per week	577	37.50
	41–48 h per week	780	50.60
	49-58 h per week	123	8.00
	≥59 h per week	60	3.90
Number of night shift	0	181	11.80
	1–4 times per month	239	15.50
	5–8 times per month	659	42.80
	>8 times per month	461	29.90
Monthly income	<4,000 yuan/month	57	3.70
	4,000 ~ 5,999 yuan/mont	149	9.70
	6,000 ~ 7,999 yuan/mont	250	16.20
	8,000 ~ 9,999 yuan/mont	368	23.90
	≥10,000 yuan/mont	71	46.50

RN, registered nurse.

### 3.2 Correlation analysis of dimensions

The mean scores for the main variables were shown in Table 2. Burnout showed strong positive correlations with WFEBRC, WFC, FWC, and somatic symptoms (*r* = 0.493, 0.553, 0.389, and 0.534, respectively; *p* < 0.001). Additionally, negative correlations were observed between burnout and the nursing work environment, WFEBRC, WFC, FWC, and somatic symptoms (*r* = −0.272, −0.413, −0.404, −0.364, and −0.370, respectively; *p* < 0.001), indicating significant inverse associations (Table 3).

**Table 2:** The scores of the research variables.

Variables	Min	Max	Scores ( $\bar{x}\pm s$ )
Burnout	−9	22.8	4.77 ± 6.16
Emotional exhaustion	0	30	11.3 ± 7.76
Depersonalization	0	30	8.61 ± 7.58
Personal accomplishment	0	36	13.79 ± 10.04
WFEBRC	19	95	42.48 ± 16.21
Work-to-family conflict	7	35	18.27 ± 6.88
Family-to-work conflict	12	60	24.21 ± 10.75
Nursing work environment	26	156	117.55 ± 24.60
Professional development	5	30	23.10 ± 5.26
Leadership and management	4	24	17.90 ± 4.40
Physician-nurse relationships	4	24	18.19 ± 4.10
Recognition atmosphere	3	18	14.02 ± 2.89
Professional autonomy	4	24	18.97 ± 3.74
Basic guarantees	3	18	12.75 ± 3.68
Adequate manpower	3	18	12.62 ± 3.68
Somatic symptoms	20	80	39.58 ± 13.61
Physical disorder	10	40	19.13 ± 6.87
Anxiety disorder	4	16	7.77 ± 2.91
Depression disorder	4	16	8.35 ± 2.99
Anxiety and depression disorder	2	8	4.34 ± 1.56

WFEBRC, Work-Family Behavioral Role Conflict Scale.

**Table 3:** The correlations analysis for the main study variables.

Variables	1	2	3	4	5	6
Burnout	1					
WFEBRC	0.493^**^	1				
Work-to-family conflict	0.553^**^	0.873^**^	1			
Family-to-work conflict	0.389^**^	0.950^**^	0.676^**^	1		
Nursing Work Environment	−0.272^**^	−0.413^**^	−0.404^**^	−0.364^**^	1	
Somatic symptoms	0.534^**^	0.655^**^	0.674^**^	0.557^**^	−0.370^**^	1

**P < 0.01, WFEBRC, Work-Family Behavioral Role Conflict Scale.

### 3.3 Mediating effect of WFEBRC on the relationship between burnout and somatic symptoms

As shown in Table 4, burnout significantly predicted somatic symptoms (*β* = 1.180, *t* = 24.763, *p* < 0.01). When WFEBRC, WFC, and FWC were included as mediators, burnout continued to significantly predict somatic symptoms (*β* = 0.616, 0.513, and 0.826; *t* = 13.295, 10.634, and 17.866, respectively; *p* < 0.01).

**Table 4:** Regression analysis of the mediation model of role conflict.

Variable	Model	*β*	*t*	*R*	*R^2^*	*F*
Behavior role conflict	1	1.180	24.763**	0.534	0.285	613.187**
	2	1.297	22.199**	0.493	0.243	492.789**
	3	0.616	13.295**	0.699	0.488	732.903**
		0.435	24.696**			
Work-to-family conflict	1	1.180	24.763**	0.534	0.285	613.187**
	2	0.618	26.050**	0.553	0.306	678.595**
	3	0.513	10.634**	0.701	0.491	742.144**
		1.079	24.962**			
Family-to-work conflict	1	1.180	24.763**	0.534	0.285	613.187**
	2	0.679	16.553**	0.389	0.151	273.987**
	3	0.826	17.866**	0.655	0.429	577.160**
		0.521	19.677**			

**p < 0.01. Model 1: Burnout prediction symptoms; Model 2: Burnout prediction behavior role conflict, work-to-family conflict and family-to-work conflict; Model 3: Burnout and behavior role conflict, work-to-family conflict and family-to-work conflict jointly predicting symptoms.

Furthermore, as shown in Tables 4, 5, the 95% CIs obtained via bootstrap analysis (5,000 samples) for both the direct effect and the indirect effects via WFEBRC, WFC, and FWC did not include 0. These findings indicate that WFEBRC, WFC, and FWC partially mediate the relationship between occupational burnout and somatic symptoms among emergency nurses.

**Table 5:** Tests for total, direct, and indirect effects of the main study variables.

Variable	Effect	*B*	*SE*	95%*CI*		%
				LLCI	ULCI	
Behavior role conflict	Total	1.180	0.048	1.087	1.247	
	Direct	0.616	0.046	0.525	0.707	52.20%
	Indirect	0.564	0.044	0.480	0.651	47.80%
Work-to-family conflict	Total	1.180	0.048	1.087	1.247	
	Direct	0.513	0.048	0.419	0.608	43.47%
	Indirect	0.667	0.044	0.582	0.755	56.53%
Family-to-work conflict	Total	1.180	0.048	1.087	1.247	
	Direct	0.826	0.046	0.736	0.917	70%
	Indirect	0.354	0.035	0.289	0.424	30%

SE, standard error; LLCI, lower limit confidence interval; ULCI, upper limit confidence interval.

### 3.4 Moderated mediation effect of the nursing work environment

Results in Table 6 indicate that the interaction term between burnout and the nursing work environment significantly predicted WFEBRC, WFC, and FWC (*β* = 0.007, 0.002, and 0.005; *t* = 3.647, 2.724, and 3.555, respectively; *p* < 0.01), suggesting a moderating effect.

**Table 6:** Analysis of the moderating effect of role conflict.

Variable	*β*	SE	*t*	95%*CI*		*R*	*R^2^*	*F*
				LLCI	LLCI			
Role conflict								
Constant	42.752	0.346	123.607**	42.074	43.431	0.577	0.333	255.082**
Burnout	1.106	0.057	19.257**	0.993	1.218			
Work Environment	−0.195	0.014	−13.627**	−0.223	−0.167			
Burnout × Work Environment	0.007	0.002	3.647**	0.003	0.010			
Work-to-family conflict								
Constant	18.356	0.142	129.621**	18.078	18.633	0.615	0.379	312.185**
Burnout	0.542	0.024	23.072**	0.496	0.589			
Work Environment	−0.076	0.006	−12.903**	−0.087	−0.064			
Burnout × Work Environment	0.002	0.001	2.724**	0.001	0.003			
Family-to-work conflict								
Constant	24.397	0.246	99.040**	23.913	24.880	0.479	0.230	152.626**
Burnout	0.563	0.041	13.776**	0.483	0.644			
Work Environment	−0.119	0.010	−11.716**	−0.139	−0.099			
Burnout × Work Environment	0.005	0.001	3.555**	0.002	0.007			

**P < 0.01; SE, standard error; LLCI, lower limit confidence interval; ULCI, upper limit confidence interval.

Simple slope analyses further revealed that the relationships between burnout and WFEBRC, WFC, and FWC were more pronounced in nurses with high levels of nursing work environment (simple slopes = 1.268, 0.592, and 0.676; *t* = 16.547, 18.871, and 12.385, respectively; *p* < 0.01) compared with those with low levels (simple slopes = 0.944, 0.493, and 0.451; *t* = 13.807, 17.493, and 9.262, respectively; *p* < 0.01). These results indicate that the effect of burnout on role conflict is significantly stronger under a more supportive work environment.

**Figure 1:**
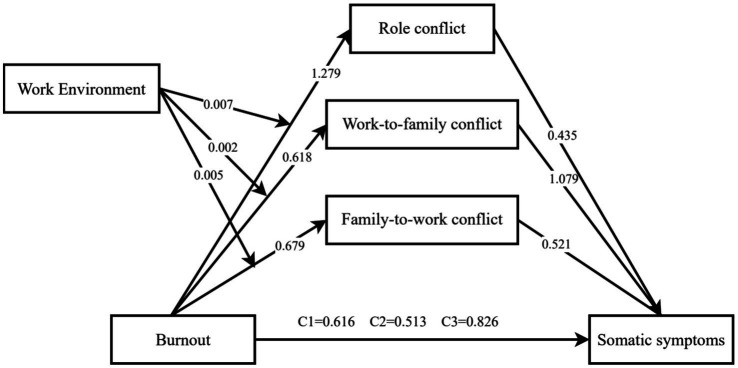
The moderated mediation model of occupational burnout on emergency nurses’ health.

**Figure 2:**
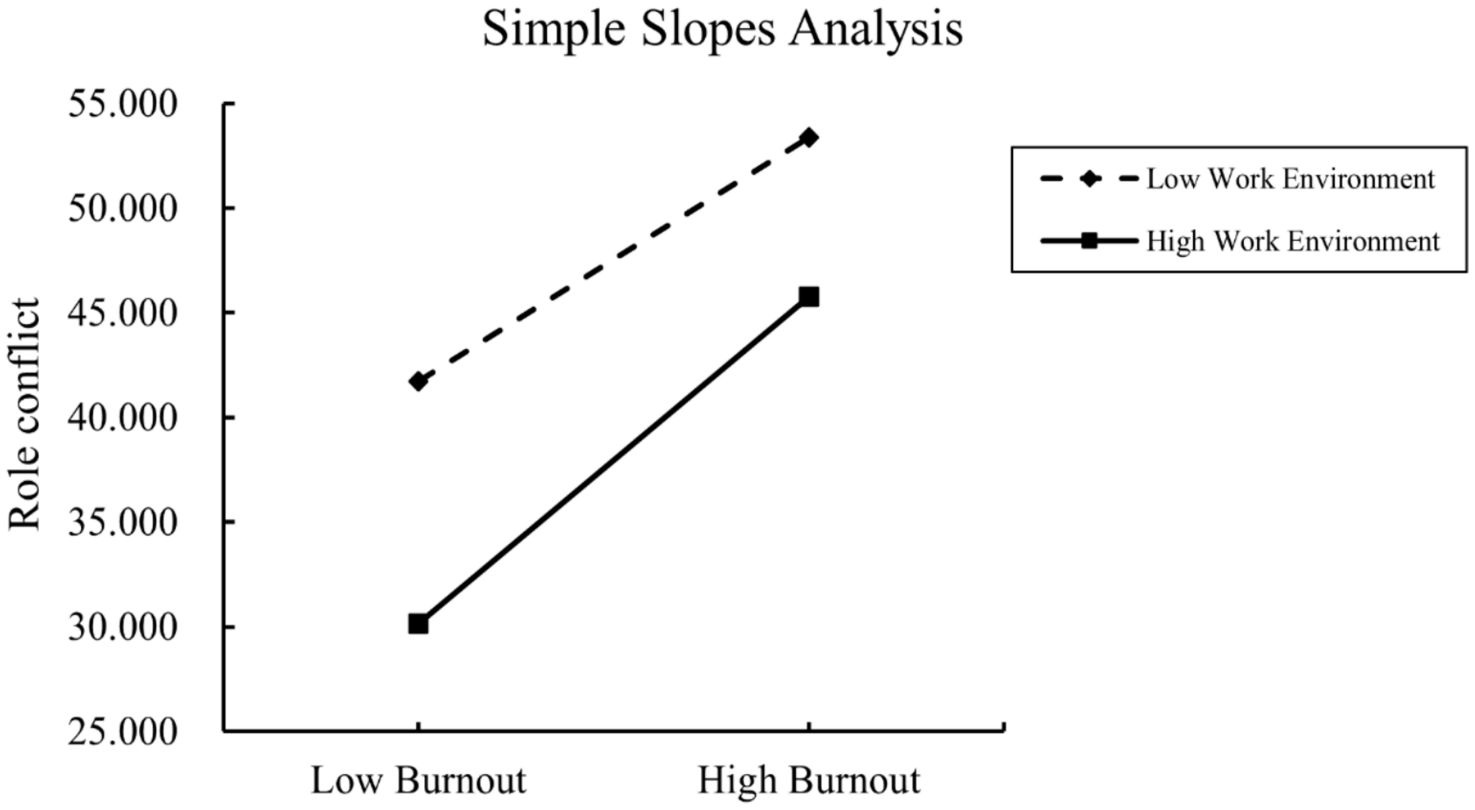
The simple slopes analysis of occupational burnout on work-family behavior role conflict moderated by different levels of working environment.

Additionally, as shown in Table 7, the indirect effects of burnout on somatic symptoms through WFEBRC, WFC, and FWC increased across three levels of the nursing work environment (*M* − 1 *SD*, *M*, and *M* + 1 *SD*). This suggests that a more favorable work environment may amplify the pathway by which burnout contributes to role conflict and, in turn, somatic symptoms (Figures 1, 2).

**Table 7:** Mediating effects at different levels of work environment.

Variable	Effect	*β*	SE	95%*CI*	
				LLCI	ULCI
Role conflict	Eff1(*M*-1SD)	0.410	0.045	0.321	0.500
	Eff2(*M*)	0.481	0.043	0.398	0.566
	Eff3(*M* + 1SD)	0.551	0.058	0.439	0.668
	Moderated mediation effects	0.003	0.001	0.001	0.005
Work-to-family conflict	Eff1(*M*-1SD)	0.532	0.048	0.439	0.624
	Eff2(*M*)	0.585	0.043	0.500	0.669
	Eff3(*M* + 1SD)	0.639	0.053	0.535	0.742
	Moderated mediation effects	0.002	0.001	0.001	0.004
Family-to-work conflict	Eff1(*M*-1SD)	0.235	0.038	0.159	0.308
	Eff2(*M*)	0.294	0.035	0.228	0.364
	Eff3(*M* + 1SD)	0.352	0.048	0.260	0.450
	Moderated mediation effects	0.002	0.001	0.001	0.005

SE, standard error; LLCI, lower limit confidence interval; ULCI, upper limit confidence interval.

## 4 Discussion

Burnout and work-family behavior role conflict have received a lot of attention in recent years in healthcare because they have a substantial impact on the health and well-being of nurses. This study found that occupational burnout negatively predicts the health of emergency nurses; that is, the more severe the occupational burnout among emergency nurses, the more pronounced their somatic symptoms and the lower their health levels. This indicates that occupational burnout has a certain degree of negative effects on both the physical and psychological symptoms of emergency nurses. This result is consistent with findings from previous research. Burnout can cause physical problems such as musculoskeletal pain, sleep disorders, and headaches (28–30). Systematic reviews have shown that burnout among emergency nurses reaches 30% and physical and mental illness are more severe than in other departments (31). Chronic occupational burnout drains the physical and mental resources of emergency nurses, causing them to suffer a dual blow to both body and mind, thereby affecting their physical and mental health.

The study’s findings, which are the first to investigate the role of conflict as a mediator between burnout and emergency nurses’ health across various work environments, support the hypothesized model. First, work-family behavior role conflict, with its two aspects, mediated the link between burnout and somatic symptoms. This study revealed that the negative effects of occupational burnout can extend beyond the workplace into the family domain. Specifically, higher levels of burnout among emergency nurses significantly increase the likelihood of work-family behavior role conflict. Such conflict exerts direct and indirect detrimental effects on nurses’ health, serving as a critical mediating mechanism through which occupational burnout impacts the well-being of emergency nurses. Ann et al. found that burnout predicted work–family conflict. Furthermore, emotional tiredness moderated the association between job expectations and work–family conflict (50). Studies found that 42.7% of emergency nurses experienced high levels of work–family conflict, with overload, frequent night shifts, and irregular working hours being significant predictors of work–family conflict among emergency nurses (18, 32). Work-family behavior role conflict modulates the association between occupational stress and physical and mental health (33–35). The repercussions of this impact mechanism begin with psychological symptoms, but as time passes, they progress to adverse effects on employees’ physical health (36–38).

In addition, WFC accounted for the biggest fraction of the total mediating effect (56.53%) among the three mediation channels identified. Work–family conflict, in particular, exacerbates burnout by disrupting the allocation of time and energy (5, 39, 40). The intense workload and extended working hours characteristic of emergency nursing frequently encroach upon nurses’ personal time, causing family disruption. Conversely, when familial pressures spill over into the workplace, the depleted psychological resources of emergency nurses cannot be adequately replenished through home support. Under these high-pressure conditions, burnout progressively accumulates. This not only impairs work performance and diminishes utilization of occupational resources, but also exacerbates work–family conflict, ultimately compromising the health of emergency nurses. A deeper knowledge of this mechanism could serve as the foundation for preventative strategies against emergency department nurse burnout as well as physical and mental health difficulties.

Another unexpected conclusion of this study is the improvement effect of a healthy work environment. This study further confirmed the buffering role of the work environment in the mediating process of ‘occupational burnout affecting emergency nurses’ health through work-family behavior role conflict.’ The results indicate that the work environment negatively moderates the first half of the pathway in the model—in other words, the interaction between the work environment and occupational burnout negatively affects work–family conflict. Burnout has a stronger moderating influence on the health of emergency nurses in a healthy work environment than in an unpleasant one. Burnout is a common occupational complication in unfavorable work situations. Compared with emergency nurses in Magnet hospitals, those in non-Magnet hospitals have higher levels of burnout and frequently work in unhealthy circumstances with insufficient nurse staffing, numerous night shifts, and excessive workloads (41). The essential characteristics of the work environment (e.g., staffing, leadership support, and resource adequacy) are critical in reducing work–family conflict. According to research, every 1-point rise in nurses’ perceived work environment scores reduces their work–family conflict scores decrease of 0.35 points (23, 42).

This finding initially appears to contradict the buffering hypothesis of the Job Demands–Resources (JD-R) model; however, it precisely reveals the complexity of the mechanisms through which job resources operate. The classic JD-R model primarily emphasizes the direct mitigating role of resources on job demands. In contrast, our results suggest that when a “healthy work environment” exists as an organizational context, it may simultaneously assume a dual role, functioning as both a resource and a demand. Specifically, the protective effect of job resources (e.g., organizational support) is subject to a threshold; beyond this critical point, an overabundance of resources may paradoxically exacerbate employees’ psychological strain (43–45).

Furthermore, when a healthy environment requires sustained employee engagement, such as maintaining harmonious interpersonal relationships or participating in team-building activities, it may encroach upon the time and psychological resources that would otherwise be allocated to recovery. This deprivation prevents already burnt-out employees from obtaining essential recuperation, thereby intensifying work–family conflict (46). In addition, a bidirectional causal relationship exists between a family-supportive environment and psychological distress: not only does the environment influence individual well-being, but individuals’ psychological distress also reciprocally shapes their perception of the environment (47). This reciprocal mechanism supports our conclusion that employees with higher levels of burnout may, driven by cognitive consistency, overemphasize the “healthiness” of their work environment (48).

Therefore, managers should recognize that when employees already exhibit symptoms of burnout, merely cultivating a harmonious atmosphere may be insufficient to alleviate the problem. Such efforts must be complemented by explicit boundary management support (e.g., prohibiting after-hours work intrusions) to transform a healthy work environment from a “hidden stressor” back into a genuine buffer.

### 4.1 Limitations

This study used a cross-sectional survey design, which involved a one-time assessment rather than a longitudinal or sequential approach. As a result, the temporal relationship between burnout and its impact on nurses’ occupational health through role conflict could not be thoroughly evaluated. Furthermore, the study focused exclusively on emergency nurses, limiting the generalizability of the findings across different clinical departments. Future studies are recommended to validate these results among nurses in diverse healthcare settings to enhance the applicability of the conclusions.

## 5 Conclusion

This study demonstrated that role conflict significantly mediates the relationship between burnout and adverse health outcomes among emergency department nurses. Moreover, the moderating role of the nursing work environment was shown to amplify this relationship, particularly under more supportive conditions. From an organizational perspective, nursing managers should prioritize interventions to reduce burnout by fostering a healthier work environment and offering innovative forms of organizational support. Effective time management is foundational for mitigating work–family conflict. In addition, seeking family support and establishing clear boundaries between professional and personal responsibilities are essential. By implementing these strategies comprehensively, nurses may alleviate burnout, achieve a better work–family balance, and, improve their physical and mental well-being.

## Data Availability

The raw data supporting the conclusions of this article will be made available by the authors, without undue reservation.
